# Evaluation of cases with hypersensitivity pneumonia: 10 year analysis

**DOI:** 10.1111/crj.13598

**Published:** 2023-02-13

**Authors:** Adem Koyuncu, Gülden Sarı, Ceprail Şimşek

**Affiliations:** ^1^ Department of Occupational Diseases, Ankara Atatürk Sanatoryum Education and Research Hospital Health Science University Ankara Turkey

**Keywords:** cryptogenic, environmental risk, hypersensitivity pneumonia, occupational risk

## Abstract

**Introduction:**

The aim of this study was to examine the clinical features of hypersensitivity pneumonia (HP) cases, diagnostic methods, and related conditions in our hospital, which is a reference clinic in Turkey for chest disease.

**Methods:**

The population of this retrospective cross‐sectional study consists of all hypersensitivity pneumonia patients followed in a tertiary hospital between 2010 and 2019. The data of 78 patients were included in the analysis. Data were grouped by source of exposure (occupational, environmental, and cryptogenic) by examining the files of the patients.

**Results:**

Occupational risk factors were detected in 29 (37.2%) of the cases, environmental risk factors were found in 24 (30.8%) cases, neither occupational nor environmental risk factors were detected in 25 (32%) cases, and they were evaluated as cryptogenic. The time from the onset of symptoms to diagnosis was 15.8 ± 26.6 months. The time from the onset of symptoms to diagnosis was found to be longer in the group with occupational risk factors compared with the other groups and was statistically significant (0.044).

**Conclusion:**

HP is a immune‐mediated interstitial lung disease induced by repeated exposure to environmental and occupational antigens. Etiological agent can be detected in HP patients by detailed questioning of occupational and environmental exposure that may be associated with the onset of symptoms in cases with suspected HP.

## Graphical abstract

The aim of this study was to examine the clinical features of hypersensitivity pneumonia cases, diagnostic methods, and related conditions in our hospital, which is a reference clinic in Turkey for chest disease. HP is a common immune‐mediated interstitial lung disease induced by repeated exposure to environmental and occupational antigens. Etiological agent can be detected in HP patients by detailed questioning of occupational and environmental exposure that may be associated with the onset of symptoms in cases with suspected HP.

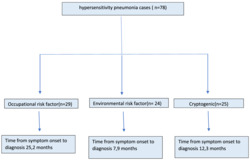

## 1 INTRODUCTION

Hypersensitivity pneumonia (HP), also called extrinsic allergic alveolitis, is an interstitial lung disease caused by repeated inhalation exposure to various antigens.^1^ HP is a syndrome involving the lung parenchyma and specifically the alveoli, terminal bronchioles, and alveolar interstitium due to a delayed allergic reaction. This reaction occurs secondary to repeated and prolonged inhalation of different types of organic dusts or other substances to which the patient is hypersensitive, especially organic dusts of animal or vegetable origin, less commonly chemicals.^2^ More than 300 etiologic agents have been identified as the cause of the disease. Bacteria, fungi, animal proteins, plant proteins, low molecular weight chemicals, and metals have been identified as inciting agents.^3^ Many inciting agents have been associated with HP since its recognition in 1700, but antigen and exposure were not identified in up to 60% of HP patients despite an extensive history.^4^ The most common forms are bird fancier's disease and farmer's lung.^5^ Due to the change in working conditions in industrialized countries, prevalence of farmer's lung decreased but HP cases due to metalworking fluids increased.^6^

Although the prevalence of HP is thought to be low, the fact that mild or subclinical HP can escape detection or be misdiagnosed as other disease or asthma, either of which may have nonspecific clinical findings that mimic HP. This suggests that the disease actually exists at a higher rate than expected. In a study, the one‐year prevalence rate of the disease was determined as 1.67–2.71 (11.2% over 65 years old) per hundred thousand people, half of the cases were evaluated as chronic HP.^7^ In the United Kingdom, the incidence was found to be 0.9 per 100 000 people in the period 1991–2003.^8^ Previously, HP was classified according to the duration of the disease at the time of admission (i.e., acute, subacute, or chronic). These categories have been vaguely defined in the current literature and have not been consistently associated with the results. It has recently been proposed to classify patients as fibrotic HP or non‐fibrotic HP based on the predominant presence or absence of radiological and/or histopathological fibrosis.^4^

Definitive diagnosis of HP is not easy due to the lack of a gold standard of diagnostic tests. The diagnosis relies on the clinical evaluation of a number of features, including a history of significant exposure to potentially causative antigens, detection of precipitating antibodies, excluding other possible interstitial lung diseases, clinical features, bronchoalveolar lavage (BAL), radiology, and pathology.^9^, ^10^ Although expert consensus statements have been published recently, there is no standardized diagnostic method and criteria. Centers tend to diagnose patients based on their own experience and consensus with their multidisciplinary teams.^11^

Avoiding the responsible antigens before occurring irreversible radiological and physical damage is the most important step in the treatment of HP. Corticosteroids and immunosuppressive drugs can be used in pharmacological treatment. Recently, antifibrotic treatments can be used depending on HP phenotypes. Lung transplantation may be considered in patients with severe clinical and functional loss.^12^

The epidemiology of HP in Turkey is not fully known. Most of the studies about HP in Turkey are in the form of case reports or case series. In a study conducted in 2010, when the population of that period in which the cases reported in Turkey were examined, it was observed that the number of cases was limited, and it was concluded that many cases were missed in terms of occupational diseases.^13^ The aim of this study was to identify causative factors of HP and develop diagnostic recommendations for these patients, in our hospital, which is a reference clinic in Turkey for chest disease. So that, the patients diagnosed as HP in Health Sciences University, Ankara Atatürk Chest Diseases and Thoracic Surgery Training and Research Hospital were examined for their clinical features, diagnostic methods, and associated conditions.

## 2 MATERIALS AND METHODS

The population of this retrospective cross‐sectional study consists of all HP patients followed in Ankara Atatürk Chest Diseases and Thoracic Surgery Training and Research Hospital, outpatient clinic and inpatient service between 1 January 2010 and 31 December 2019. It is planned to reach the entire population without sampling. The study was started after the approval of the ethics committee. Eighty patients diagnosed with J67 (hypersensitivity pneumonitis, due to organic dust) and J67 sub diagnostic codes between 2010 and 2020 in the hospital management system were included in the study. According to ICD 10 classification, J67.0 Farmer lung, J67.1 Bagassosis, J67.2 Bird fancier lung, J67.3 Suberosis, J67.4 Maltworker lung, J67.5 Mushroom‐worker lung, J67.6 Maple‐bark‐stripper lung, J67.7 Air‐conditioner and humidifier lung, J67.8 Hypersensitivity pneumonitis due to other organic dusts, and J67.9 Hypersensitivity pneumonitis due to unspecified organic dust were included in the study. Two patients whose information was missing in the hospital information management system were excluded from the study, and the data of 78 patients were included in the analysis.

For the research, the data of the cases diagnosed with HP, laboratory test results in the hospital information management system, respiratory function evaluation results, and radiological images were used. Data were grouped by source of exposure (occupational, environmental, and cryptogenic) by examining the files of the patients. Occupational risk factor group; In which agents that can cause HP are detected those who have in the workplace. Environmental risk factor group; in which the agent that can cause HP is detected in the environment they live in. Cryptogenic group; In those who do not have a history of occupational or environmental risk factors that may cause HP. In the literature, if an inducer agent for HP is not detected, it is recommended to call it cryptogenic HP.^10^ The dependent variable of the study was the condition causing hypersensitivity pneumonitis (occupational risk factor, environmental risk factor, and cryptogenic), and its independent variables were socio‐demographic (age and gender) data and characteristics related to working life, functional evaluation results (forced expiratory volume in the first second [FEV1], forced vital capacity [FVC], FEV1/FVC ratio, diffusing capacity of the lung for carbon monoxide [DLCO]), laboratory results (white blood cell [WBC], lactate dehydrogenase [LDH], C reactive protein [CRP], sedimentation), diagnostic methods, exposure characteristics, and radiological evaluation results (high‐resolution computed tomography [HRCT]). In the study, a patient follow‐up form was used as a data collection tool.

Statistical analyses were performed using the SPSS 22.0 (standard version) package program. Descriptive statistics for continuous variables were expressed as mean ± standard deviation (SD) and minimum and maximum values, whereas for categorical variables, it was expressed as numbers and percentages. Independent groups' *t*‐test was used to compare two‐group continuous variables. Chi‐square test was used to compare categorical variables. One‐way analysis of variance (one‐way ANOVA) was performed to determine whether there was a difference between the means for the continuous variables according to the categorical variables. For all statistical calculations, *p* < 0.05 was considered statistically significant.

## 3 RESULTS

The research group consists of 78 cases diagnosed with HP between 1 January 2010 and 31 December 2019 in Ankara Atatürk Chest Diseases and Thoracic Surgery Training and Research Hospital. Our study included 78 patients with HP, and the majority of the group consisted of men. There were 41 men (52.6%) and 37 women (47.4%). The mean age of the patients at diagnosis was 49.36 ± 14.0 (20–76). Occupational risk factors were detected in 29 (37.2%) of the cases, environmental risk factors were found in 24 (30.8%) cases, neither occupational nor environmental risk factors were detected in 25 (32%) cases, and they were evaluated as cryptogenic. While the agent that could cause HP could not be detected in 25 (32.1%) of the cases, mold or fungus elements in nine (11.5%), chemical agents in 14 (17.9%), and animal proteins in 30 (38.5%) were responsible as an independent risk factor for progression into HP. Of the patients, 22 (28.2%) were smokers, 48.7% had never smoked, and 23.1% had smoked before. The mean duration of smoking among smokers was 20.9 ± 12.6 years. The time from the onset of symptoms to diagnosis was 15.8 ± 26.6 months (0.25–120). Pigeon‐specific antibodies were studied in three patients, and it was positive in one patient (Table 1).

**TABLE 1:** Some demographic characteristics of the cases.

		*n*	%
Gender (*n* = 78)	Male	41	52.6
	Female	37	47.4
Age (mean ± SD), years			49.36 ± 14.0 (20–76)
Risk factor (*n* = 78)	Occupational risk factor	29	37.2
	Environmental risk factor	24	30.8
	Cryptogenic	25	32.0
Agent that can cause (*n* = 78)	Mold or fungus	9	11.5
	Chemical agent	14	17.9
	Animal protein	30	38.5
	Unknown	25	32.1
Smoking (*n* = 78)	Smoker	22	28.2
	Ex smoker	18	23.1
	Nonsmoker	38	48.7
Smoking duration (mean ± SD), years			20.9 ± 12.6
Shortness of breath (*n* = 77)	Yes	73	94.8
	None	4	5.2
Cough (*n* = 77)	Yes	61	79.2
	None	16	20.8
Fever (*n* = 77)	Yes	4	5.2
	No	73	94.8
Weight loss (*n* = 77)	Yes	6	7.8
	No	71	92.2
Clubbing (*n* = 77)	Yes	7	9.1
	No	70	90.9
Rall (*n* = 77)	Yes	26	33.8
	No	51	66.2
Time from symptom onset to diagnosis (mean ± SD), months			15.8 ± 26.6 (0.25–120)
Pigeon specific antibody (*n* = 3)	Positive	1	33.3
	Negative	2	66.7

Abbreviation: SD, standard deviation.

While 73 (94.8%) of the cases had shortness of breath and 61 (79.2%) had cough, fever and weight loss were rare symptoms. On physical examination, clubbing was found in approximately one in 10 patients and rall in approximately one third (33.3%) of the patients (Table 1).

Laboratory tests and pulmonary function test results are given in Table 2. BAL examination was performed in 45 (57.7%) of the cases. In the BAL fluid, the mean lymphocyte was 28.6 ± 17.7 (1.2–70), whereas the mean CD4/CD8 ratio was 1.3 ± 1.2 (0.19–5.90).

**TABLE 2:** Laboratory and pulmonary function test results of the cases.

White blood cell (WBC) (mean ± SD) *n* = 78	9.11 ± 2.7 (3.9–17.4)
C reactive protein (CRP) (mean ± SD) *n* = 78	18.8 ± 40 (0.14–240)
Sedimentation (mean ± SD) *n* = 78	30.5 ± 21.0 (2–106)
Lactate dehydrogenase (LDH) (mean ± SD) *n* = 78	255.5 ± 104.5 (108–701)
Ratio of lymphocytes (*n* = 45)	28.6 ± 17.7 (1.2–70)
CD4/CD8 ratio (*n* = 45)	1.3 ± 1.2 (0.19–5.90)
FVC (mean ± SD) *n* = 66	74.0 ± 18.0 (20–118)
FEV1 (mean ± SD) *n* = 66	70.6 ± 16.6 (29–115)
FEV1/FVC ratio (mean ± SD) *n* = 66	84.2 ± 10.9 (35–107)
DLCO (mean ± SD) *n* = 43	88.5 ± 24.8 (40–148)

Abbreviations: DLCO, diffusing capacity of the lung for carbon monoxide; FEV1, forced expiratory volume in the first second; FVC, forced vital capacity; SD, standard deviation.

All patients had computed tomography of the thorax. The most common finding in tomography findings was reticulation (89.7%), followed by ground glass (85.9%), nodule (74.4%), emphysema (56.4%), and honeycomb (19.2%). Radiographically, approximately one fifth (20.5%) of the cases had fibrosis findings (Table 3).

**TABLE 3:** Computed tomography findings of the cases.

		*N*	%
Reticulation	Yes	70	89.7
	No	8	10.3
Ground glass opacity	Yes	67	85.9
	No	11	14.1
Nodule	Yes	58	74.4
	No	20	25.6
Emphysema	Yes	44	56.4
	No	34	43.6
Honeycombing	Yes	15	19.2
	No	63	80.8
Fibrosis	Yes	16	20.5
	No	62	79.5

Biopsy was performed in 43 (55.1%) of the patients, open lung biopsy was performed in more than half of the patients (53.4%) who underwent biopsy. Transbronchial lung biopsy was performed in 20 patients (46.5%), surgical lung biopsy in 10 patients (23.3%), and both transbronchial and surgical biopsies in 13 patients (30.2%). According to the pathology results, fibrosis was not detected in any of the patients who underwent transbronchial biopsy, and fibrosis was found in 17 of the patients who underwent surgical biopsy. Steroid treatment was administered to approximately three quarters (71.4%) of the patients. HP was diagnosed with the decision of the multidisciplinary council in approximately one fourth of the patients (24.7%) (Table 4).

**TABLE 4:** Some diagnostic and treatment features of the cases.

		*N*	%
Biopsy (*n* = 78)	Yes	43	55.1
	No	35	44.9
Biopsy type (*n* = 43)	Transbronchial forceps	20	46.5
	Surgical lung biopsy	10	23.3
	Transbronchial forceps + surgical lung biopsy	13	30.2
Treatment administered (*n* = 77)	Drug‐free follow‐up	16	20.8
	Corticosteroids	55	71.4
	Corticosteroids + other treatments	6	7.8
Multidisciplinary council (*n* = 78)	Yes	19	24.7
	No	58	75.3
Classification	Non‐fibrotic	50	64.1
	Fibrotic	28	35.9

The patients were divided into two groups according to the presence and absence of fibrosis radiologically and pathologically. There were 50 non‐fibrotic (64.1%) and 28 fibrotic (35.9%) cases. Of those with fibrotic disease, 16 had radiological and 17 pathological fibrosis, and five had both radiological and pathological fibrosis.

No statistically significant difference was found in the non‐fibrotic and fibrotic groups in terms of gender, causative agent, FVC in pulmonary function test, and time from symptom onset to diagnosis. The mean age of the non‐fibrotic patient group was lower, and the lymphocyte ratio was statistically significantly higher (*p* = 0.002, *p* = 0.014). While clubbing was not seen at all in the non‐fibrotic patient group, it was found to be statistically significantly higher in the fibrotic group (*p* < 0.001) (Table 5).

**TABLE 5:** Relationship between non‐fibrotic and fibrotic classification of HP and some variables.

		Non‐fibrotic HP		Fibrotic HP		*p*
		*n*	%	*n*	%	
Gender	Female	24	48.0	13	46.4	0.541^a^
	Male	26	52.0	15	53.6	
Causative agent	Known	36	72.0	16	59.2	0.188^a^
	Unknown	14	28.0	11	40.8	
Clubbing	Exists	0	0	7	25.9	**<0.001** ^a^
	Non‐exists	50	100	20	74.1	
Age (mean ± SD), years		45.88 ± 13.8		55.57 ± 12.2		**0.002** ^b^
Ratio of lymphocytes		33.04 ± 17.3		19.86 ± 15.3		**0.014** ^b^
Forced vital capacity (FVC) (%) (mean ± SD)		75.31 ± 17.9		72.08 ± 18.4		0.484^b^
Time from symptom onset to diagnosis (mean ± SD), months		14.89 ± 26.9		16.91 ± 26.55		0.747^b^

Abbreviations: HP, hypersensitivity pneumonia; SD, standard deviation.

^a^ Chi‐square test.

^b^ Independent groups *t*‐test.

Although there was no statistically significant difference in the age at diagnosis of the patients in the occupational risk factor, environmental risk factor, and cryptogenic group, the mean age of the patients with occupational risk factors was lower than the other two groups. There was no statistically significant difference between these three groups in terms of duration of smoking and pulmonary function test findings. In the cryptogenic patient group, the lymphocyte ratio and WBC count in BAL fluid were found to be statistically significantly lower than the other groups (*p* = 0.045, *p* = 0.038). The time from the onset of symptoms to diagnosis was found to be longer in the group with occupational risk factors compared with the other groups and was statistically significant (0.044) (Table 6).

**TABLE 6:** Relationship between exposure characteristics and some variables.

		*N*	Mean	SD	Minimum	Maximum	*p*
Age at diagnosis	Occupational risk factor	29	45.59	13.8	20	75	0.152
	Environmental risk factor	24	52.96	13.6	29	76	
	Cryptogenic	25	50.28	14.1	25	76	
Smoking (pack/year)	Occupational risk factor	10	22.00	14.5	1	40	0.731
	Environmental risk factor	6	17.33	10.1	6	30	
	Cryptogenic	6	22.75	12.8	7.5	40	
Ratio of lymphocytes	Occupational risk factor	19	30.34a	17.8	1.5	70	**0.045**
	Environmental risk factor	13	35.74a	17.7	9.9	64	
	Cryptogenic	13	19.0b	14.2	1.2	44	
Forced expiratory volume in the first second (FEV1)	Occupational risk factor	26	70.11	14.4	29	93	0.603
	Environmental risk factor	18	73.94	20.0	36	115	
	Cryptogenic	22	68.68	16.5	33	91	
White blood cell (WBC)	Occupational risk factor	29	8.5a	2.2	5.4	17.4	**0.038**
	Environmental risk factor	24	8.6a	2.5	3.9	15.2	
	Cryptogenic	25	10.2b	3.2	4.8	17.4	
Time from onset of symptoms to diagnosis (month)	Occupational risk factor	29	25.2b	33.7	0.25	120	**0.044**
	Environmental risk factor	24	7.9a	13.3	1	60	
	Cryptogenic	24	12.3a	24.2	1	120	

*Note*: One way analysis of variance (ANOVA) (post hoc Tukey); a,b: the difference between groups that do not have the same letter in each line is significant (*p* < 0.05).

Abbreviations: SD, standard deviation; WBC, white blood cell.

## 4 DISCUSSION

In our study, the socio‐demographic characteristics, diagnosis, and treatment characteristics of 78 cases diagnosed with HP were evaluated. The number of male cases was found to be higher than females. In a cohort analysis performed on the epidemiology of hypersensitivity pneumonitis in the United States, 58% of patients were identified as women; in previous studies, there was an excess of men, and in this study, the reason for the excess of women was considered as the fact that women began to use health services more.^7^ In the population‐based HP incidence study in Denmark, it is reported that the disease is dominant among males, similar to our study (57%). While a relatively gender‐balanced prevalence emerges in HP, it may differ by gender, particularly as exposure to certain etiologic antigens in occupational HP may be gender‐based. Men appear to be more likely than women to develop HP due to exposure to agricultural and metalworking fluids.^14^ In our study, the mean age of the patients diagnosed with HP was 49.36 ± 14.0 years, and the minimum age of diagnosis was 20, and the highest age of diagnosis was 76. In the study of Kumar et al.^15^ in India with 103 HP patients, the mean age was found to be 47 ± 12.8 years, and it was found to be similar to our study. In the study performed by Santos et al.^16^ in Portugal, where the characteristics of 209 HP patients were evaluated, the mean age was determined as 58.3 ± 16.0 years, and the mean age was found to be slightly higher than our study. When the agents that could cause the disease were examined in the files of the patients, no risk factors were found in approximately one third of the patients in our study. In a study conducted in England, the cause could not be determined in 49% of the cases, despite detailed evaluation of exposure histories, laboratory, and radiological findings.^6^ Among the inducer factors detected in our study, the most frequent exposure was animal proteins with 38.5%. In the study by Kumar et al.,^15^ 61% of the patients had a history of exposure to an inducer agent, and pigeon exposure was the most common (56%) of them. Fernández Pérez et al.^7^ showed that the indetermination of the inducer agent was associated with shorter survival in patients with chronic HP. By questioning the occupational and environmental exposure that may be associated with the onset of symptoms in cases with suspected HP, the etiological agent can be detected. After diagnosis, antigen avoidance is the rule whenever possible before permanent radiological and physical damage occurs, and recovery can be achieved without the need for additional steroid therapy.

About a quarter of the patients who diagnosed with HP were smokers. Unlike other lung diseases, HP is seen in 80%–95% non‐smokers.^17^ Although the reasons and mechanism of it are less common in smokers, it is thought that the anti‐inflammatory effect of nicotine may play a role.^18^

Common symptoms and signs of HP include shortness of breath, cough, and mid‐inspiratory squeaks. Less frequently, there may be constitutional symptoms such as weight loss, flu‐like symptoms (chills, low‐grade fever, and malaise), chest tightness, and wheezing, as well as physical examination findings such as rales and cyanosis.^4^ Consistent with the literature, the most common symptoms of patients in our study group were dyspnea and cough. HP should be considered in the differential diagnosis of patients with unexplained shortness of breath and cough, and detailed occupational and environmental exposures should be questioned.^19^

HP is a difficult disease to diagnose, and in our study, the mean time from the onset of the first symptoms of the patients to the diagnosis was 15.8 months, whereas this was 12 months (2 months–30 years) in the study of Szturmowicz et al.^20^ Shobeirian et al.^21^ found this time as 7.47 (1–28) years in their study. In the study of Kumar et al.,^15^ it was determined that 86% of HP patients had symptoms for more than 6 months. While questioning the patients about the HP risk factors, clinicians firstly think about the environmental risk factors. When a risk factor that can cause HP is detected, it becomes easier to diagnose the disease. In our study, the mean time from the onset of the first symptoms to diagnosis was 7.9 months in the group with environmental risk factors, whereas this period was 25.2 months in the group with occupational risk factors. So that the possible exposures of the patients in the working environment should be evaluated in detail.

BAL is the most sensitive tool to detect alveolitis in patients with suspected HP, and in HP patients, BAL usually detects lymphocyte‐rich alveolitis. The lymphocyte count is usually greater than 50% in subacute HP.^9^ In our study group, the lymphocyte rate in BAL was found to be over 30% in the groups with occupational and environmental risk factors, whereas the lymphocyte rate was 19% in the cryptogenic patient group. When both the lymphocyte rate is determined above 30% and occupational and environmental risk factors are detected, the diagnosis of HP can be made more easily.

Pulmonary function abnormalities in HP play an important role in determining the severity of the disease, but they do not differentiate from other interstitial lung diseases. They are useful for evaluating physiological abnormalities and associated impairment. Restrictive pathology can be seen in HP patients. However, a combined obstructive/restrictive functional pattern may be observed in some patients, as HP may affect the small airways. An early reduction in DLCO can often be found.^22^ While a mild decrease in FEV1, FVC, and diffusion was found in the mean respiratory function tests of the patients in our study, the FEV1/FVC ratio was found to be normal.

Posterior anterior chest X‐ray has limited contribution in diagnosing HP, but it is thought to have clinical importance in excluding other diagnoses. In 20% of HP patients, no abnormality may be detected in the chest X‐ray. It is often seen as nodular or reticulonodular ground‐glass opacities with preservation of the lower lung areas on chest X‐ray.^23^ HRCT is widely used in the diagnosis and follow‐up of patients and in determining the progression and prognosis of the disease.^21^ Centrilobular ground glass nodules are usually seen in the middle and upper lung zones in subacute HP. All CT patterns are seen in fibrotic HP, with or without honeycombing, including reticulation, traction bronchiectasis, and volume depletion. They often show a predominant distribution of fibrosis in the upper lobe, but diffuse and lower lobe predominant changes have also been described.^24^ The presence and extent of fibrotic structures are important because the extent of pulmonary fibrosis is positively correlated with increased mortality in patients with HP. In our study group, the most common HRCT findings were reticulation (89.7%), ground glass opacity (85.9%), and nodules (74.4%), and fibrosis was detected in approximately one fifth of the patients. In the study of Shobeirian et al.,^21^ similar to our study, reticulation and ground glass opacities were detected in 93% of the patients, whereas fibrosis was detected in 75% of the patients.

The presence of radiographic or histopathological fibrosis is the primary determinant of prognosis, the   American Thoracic Society (ATS), the Japanese Respiratory Society (JRS), and the Asociación Latinoamericana del Tórax (ALAT) guideline categorizes HP as either nonfibrotic or fibrotic.^4^ This category was considered to be more consistently associated with the clinical course, outcomes, and treatment efficiency. Nishida et al. evaluated 61.1% of 121 HP patients as non‐fibrotic in their retrospective review; similarly, we demonstrated the non‐fibrotic patients group as 64.1% among the study group. According to the ATS/JRS/ALAT guideline, patients with fibrotic HP are more likely to be older, have an unidentified inciting agent, and have a lower vital capacity (VC), diffusion capacity, and percentage of lymphocytes in their BAL fluid than patients with nonfibrotic HP. In our study, there was no statistically significant difference in terms of gender, causative agent, FVC in pulmonary function test, and time from the onset of symptoms to diagnosis, however the fibrotic patient group was older, has lower lymphocyte rates in their BAL fluid, and has the most clubbing.^4^ In the study of Hill et al., fibrotic patients were slightly older, with no difference in gender, smoking history, solicited frequency of exposure, or serology findings. Fibrotic patients also had lower pulmonary function findings compared with non‐fibrotic patients. Similar to our findings, the lymphocyte ratio in BAL was 19.18% in the fibrotic group, whereas it was 29.23% in the nonfibrotic group.^25^ According to Dabir et al. stated that VC and diffusion capacity decrease in both fibrotic and non‐fibrotic types, and decrease more, older, lymphocyte ratio lower, unknown antigen more common in fibrotic type.^26^ Lacasse et al. found that when classifying patients based on a combination of clinical features, imaging, and pathophysiology, those with a sub‐acute presentation were particularly difficult to define and that a two‐category model was a better fit. Cluster 1 comprised patients with recurrent and systemic symptoms including chest tightness, shortly after antigen exposure, with a greater likelihood of normal imaging, and Cluster 2 comprised advanced fibrotic disease with inspiratory crackles, clubbing, and low lung function. In our study, statistically significant clubbing was found in the fibrotic group, similar to the study of Lacasse et al.^27^

There is no single, definitive gold standard test for the diagnosis of HP. Three main points are emphasized for HP diagnostic criteria, these are identification of repeated exposure to antigens, radiological pattern, and lymphocytosis/histopathological findings in BAL. A multidisciplinary team discussion consisting of a radiologist and a pathologist is recommended for diagnosing HP.^4^ One fourth of the patients diagnosed in our study were diagnosed with HP with the decision of a multidisciplinary council consisting of a radiologist, pathologist, and thoracic surgeon as recommended in the guidelines.

In our study group, 20% of the patients were followed up with drug‐free treatment, whereas 71% were using steroids and 7% were using immunosuppressive drugs in addition to steroids. The first treatment intervention in HP should be the complete prevention of the patient's exposure to the inducer agent from the work environment and living area.

Patients should be warned against further exposure to known inducers of HP, including quilts, pillows, ventilation systems, ovens, air conditioners, and latent antigens in filters. The first‐line medical treatment is systemic steroids. Although the general goal is to give the lowest possible dose and the shortest duration of treatment, no study has been able to determine the exact duration of treatment yet. It starts with 0.5 mg/kg and is used in varying doses and times over a long period of time, from a few months to a few years. Additional immunosuppressive agents may be considered in chronic HP patients, especially those with progressive disease.^10^

The current study has limitations. First, it is a single‐center, retrospective study. Second, the records of the patients were accessed from their files, and finally, only the diagnosed patients were included in the study. The hospital is a third‐level reference hospital, it accepts patients from many parts of the country. After the patients are diagnosed, some of the patients are treated in local hospitals where they live. Therefore, we could not evaluate the patients' prognosis. Although HP has a low prevalence, we have a high number of patients due to the fact that it was performed in the 3rd level reference hospital, the occupational anamnesis of the patients due to the occupational disease clinic in the hospital, and the evaluation of the patients with a multidisciplinary council are the strengths of our study. A prospective, multicenter, and large sample cohort study in Turkey would be useful to understand the incidence in our country, clinical diagnosis accuracy, management, and prognosis of HP.

In summary, HP is a common immune‐mediated interstitial lung disease induced by repeated exposure to environmental and occupational antigens. HP should be considered in the differential diagnosis in patients with unexplained dyspnea and cough. In our study, the mean time from the onset of the first symptoms of the patients to the diagnosis was 15.8 months. In the group with occupational risk factors, the time from the onset of symptoms to diagnosis was 25.2 months. Etiological agent can be detected in HP patients by detailed questioning of occupational and environmental exposure that may be associated with the onset of symptoms in cases with suspected HP, and by removing this etiological agent, recovery can be achieved without detecting functional loss by avoiding the responsible antigens before permanent radiological and physical damage occurs. Therefore, it is very important to identify the inciting antigen and avoid exposure to improve the prognosis of HP.

## AUTHOR CONTRIBUTIONS

Aden Koyuncu and Ceprail Şimşek designed the study. Aden Koyuncu and Gülden Sarı performed the study. Adem Koyuncu and Gülden Sarı collected the data. Adem Koyuncu analyzed the data. Adem Koyuncu and Ceprail Şimşek wrote the first draft of the manuscript. All authors have read and approved the final manuscript.

## CONFLICT OF INTEREST STATEMENT

This study has no potential conflict of interest with any institution or individual. The authors declare that they have no competing interests.

## ETHICS STATEMENT

Ethics committee approval of the study was obtained with the decision numbered 139/2021 of Ankara Keçiören Training and Research Hospital Ethics Committee.

## Data Availability

Data from this trial are available upon reasonable request and approval by the corresponding author.
